# *clusterMaker: *a multi-algorithm clustering plugin for Cytoscape

**DOI:** 10.1186/1471-2105-12-436

**Published:** 2011-11-09

**Authors:** John H Morris, Leonard Apeltsin, Aaron M Newman, Jan Baumbach, Tobias Wittkop, Gang Su, Gary D Bader, Thomas E Ferrin

**Affiliations:** 1 Department of Pharmaceutical Chemistry, University of California San Francisco, San Francisco, California, USA; 2 Institute for Stem Cell Biology and Regenerative Medicine, Stanford University School of Medicine, Stanford, California, USA; 3 Max Planck Institute for Informatics, Saarbrücken, Germany; 4 Buck Institute for Age Research, Novato, California, USA; 5 Bioinformatics Program, University of Michigan, Ann Arbor, MI, USA; 6 National Center for Integrative Biomedical Informatics, University of Michigan, Ann Arbor, MI, USA; 7 The Donnelly Centre, University of Toronto, Toronto, Ontario, Canada; 8 Department of Molecular Genetics, University of Toronto, Ontario, Canada; 9 Department of Bioengineering and Therapeutic Sciences, University of California San Francisco, San Francisco, California, USA

## Abstract

**Background:**

In the post-genomic era, the rapid increase in high-throughput data calls for computational tools capable of integrating data of diverse types and facilitating recognition of biologically meaningful patterns within them. For example, protein-protein interaction data sets have been clustered to identify stable complexes, but scientists lack easily accessible tools to facilitate combined analyses of multiple data sets from different types of experiments. Here we present *clusterMaker*, a Cytoscape plugin that implements several clustering algorithms and provides network, dendrogram, and heat map views of the results. The Cytoscape network is linked to all of the other views, so that a selection in one is immediately reflected in the others. *clusterMaker *is the first Cytoscape plugin to implement such a wide variety of clustering algorithms and visualizations, including the only implementations of hierarchical clustering, dendrogram plus heat map visualization (tree view), k-means, k-medoid, SCPS, AutoSOME, and native (Java) MCL.

**Results:**

Results are presented in the form of three scenarios of use: analysis of protein expression data using a recently published mouse interactome and a mouse microarray data set of nearly one hundred diverse cell/tissue types; the identification of protein complexes in the yeast *Saccharomyces cerevisiae*; and the cluster analysis of the vicinal oxygen chelate (VOC) enzyme superfamily. For scenario one, we explore functionally enriched mouse interactomes specific to particular cellular phenotypes and apply fuzzy clustering. For scenario two, we explore the prefoldin complex in detail using both physical and genetic interaction clusters. For scenario three, we explore the possible annotation of a protein as a methylmalonyl-CoA epimerase within the VOC superfamily. Cytoscape session files for all three scenarios are provided in the Additional Files section.

**Conclusions:**

The Cytoscape plugin *clusterMaker *provides a number of clustering algorithms and visualizations that can be used independently or in combination for analysis and visualization of biological data sets, and for confirming or generating hypotheses about biological function. Several of these visualizations and algorithms are only available to Cytoscape users through the *clusterMaker *plugin. *clusterMaker *is available via the Cytoscape plugin manager.

## Background

High-throughput techniques to generate genomic, proteomic, transcriptomic, metabolomic, and interactomic data continue to advance, generating huge data sets covering more species and more information about the biology of individual species than ever before. Along with this increase in the different types and amount of data, there have been many advances in analytical techniques. One particular technique that has seen wide use in 'omics studies is clustering. Clustering algorithms detect patterns within data sets, and organize related genes, proteins, or other key elements to highlight those patterns.

**Figure 1:**
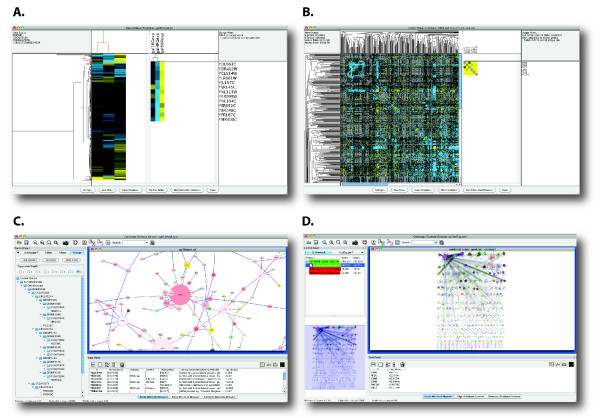
**Screenshots of *clusterMaker *visualizations**. (A) and (C) show the results of hierarchically clustering (by expression data) the yeast protein-protein interaction network included with Cytoscape (galFiltered.cys). (A) TreeView visualization showing the clustering of both nodes and attributes. (B) The symmetrical TreeView of an EMAP showing a selected cluster. (C) Cytoscape screenshot of the network used to produce (A). The group hierarchy is shown. The groups (and nodes that are part of those groups) are selected as a subtree in the TreeView. (D) The new network resulting from an MCL clustering of the TAP-MS data from Collins, et al. [52]. The option to restore inter-cluster edges after the automatic layout was selected.

One of the most familiar approaches is the hierarchical clustering of genes and their expression levels under various conditions to produce a dendrogram and heat map (Figure 1A) for analyzing and visualizing microarray data [1]. Hierarchical clustering has also been used to analyze genetic interaction data based on double-deletion mutants [2,3]. Such interaction networks can be represented as matrices of genes against genes, where each cell contains the strength of the interaction between two genes (Figure 1B).

A second clustering approach identifies stable complexes from large sets of protein-protein interactions. Such network clustering algorithms include Molecular Complex Detection (MCODE) [4], Restricted Neighborhood Search Clustering (RNSC) [5], Super Paramagnetic Clustering (SPC) [6], Markov Clustering (MCL) [7,8], and hierarchical clustering [9]. Given a protein-protein interaction network (Figure 1C), the goal is to isolate the complexes from the less stable or transient interactions (Figure 1D).

A third use of clustering is the identification of similar groups of proteins for the purpose of classification [10], that is, inferring properties of proteins of unknown function based on their similarity to proteins of known function. There are many approaches to this classification, including machine learning [11-13] (see [11] for a good overview) as well as clustering large groups of proteins based on either sequence or structural similarity metrics [7,8,14-28]. Clustering algorithms that have been applied to the categorization of proteins include Spectral Clustering of Protein Sequences (SCPS) [24], TransClust [25,29], MCL [7,8], Affinity Propagation [27], and FORCE [26].

Cytoscape [30,31] is an open-source, cross-platform software package for visualizing and analyzing biological networks. Cytoscape provides an extensive plugin application programming interface (API) that allows programmers to extend the native capabilities of Cytoscape to provide new functionality. Cytoscape currently lists over 100 plugins, many of which perform some kind of clustering. However the user interface for each of these individual plugins is very different, and there is no interaction between them.

*clusterMaker *is a new Cytoscape plugin that provides many frequently used clustering algorithms, including nearly all of the algorithms named above as well as heat map and dendrogram visualizations. The visualizations are all linked to the Cytoscape network, allowing selections in the network to be reflected in one or more of the other views, and selections in the heat maps to be reflected in the network view and all other visible heat maps. *clusterMaker *currently provides ten clustering algorithms in two broad categories, *network clustering *and *attribute clustering*, together with a unified user interface.

### Network clustering algorithms

Network clustering algorithms find densely connected regions in a network. There are multiple approaches to network clustering, including using graph algorithms to find dense regions, either using a local approach starting with a node neighborhood or using a global approach starting with the entire graph and iteratively partitioning it into clusters, and using linear algebra to operate directly on the adjacency matrix. The network clustering algorithms in *clusterMaker *are: MCL [7,8], Affinity Propagation [27], MCODE[4], Community Clustering (GLay) [32,33], SCPS [24], TransClust [25], and AutoSOME [34]. These algorithms are generally used for finding modules and complexes within protein-protein interaction networks [4,33,35,36] and for identifying functionally related groups of proteins within large protein-protein similarity networks [7,24,25,37]. *clusterMaker *also includes the Connected Components algorithm, which assigns existing network partitions (connected components) to clusters. *clusterMaker *provides the only implementations of SCPS and AutoSOME available within Cytoscape, and the only multi-threaded native Java MCL implementation.

### Attribute clustering algorithms

Attribute clustering algorithms group nodes based on similarity of their node attributes or on the basis of a single edge attribute. The attribute clustering algorithms in *clusterMaker *are: Hierarchical, k-means, k-medoid, and AutoSOME. Note that AutoSOME is in both lists, and may be used to generate networks based on node attributes.

Hierarchical, k-medoid, and k-means algorithms are commonly used for clustering gene expression data [1] and genetic interaction profiles [2]. AutoSOME is typically used for clustering expression data and general network partitioning. In general, however, most of the clustering algorithms may be used for either purpose provided the data is transformed appropriately. *clusterMaker *provides the only implementation of these clustering algorithms in Cytoscape. In addition to the basic k-means and k-medoid algorithms, beginning with version 1.10, *clusterMaker *provides the facility to choose *k *by finding the *k *that maximizes the average silhouette for the solution [38]. Coupled with *clusterMaker's *heatmap and dendrogram visualization, this represents a reasonably complete clustering environment for the analysis and visualization of expression profiles and other microarray experiments within the context of pathway, protein-protein interaction, and other network-oriented biological data.

## Implementation

*clusterMaker *is implemented as a plugin to the open source network analysis and visualization package, Cytoscape [30]. *clusterMaker *extends Cytoscape's capabilities by providing various clustering algorithms and associated visualizations, and intuitively links those to the network visualization provided by Cytoscape. *clusterMaker *is written entirely in Java to allow easy portability to any platform supporting the Java virtual machine.

*clusterMaker *exposes parameters for each clustering algorithm via a graphical user interface (GUI). When a user selects an algorithm, a dialog appears for specifying the node or edge attribute(s) to use for the data source, along with any algorithm-specific parameters such as *k *for k-means clustering, the expansion factor for MCL, the linkage for hierarchical clustering, and the distance metric for k-means, k-medoid, or hierarchical clustering. For example, the k-means, k-medoid, and hierarchical implementations support clustering on both genes (nodes) and arrays (attributes). A typical application might be to select a set of node attributes containing the expression change ratios for different time points or conditions compared to a control, and then perform hierarchical clustering on the nodes and (optionally) attributes. All of the clustering methods allow selection of a single edge numeric attribute for clustering. For k-means, k-medoid, and hierarchical clustering, this attribute is used to construct a symmetric adjacency matrix for clustering. For network clustering algorithms, the edge weight is assumed to be a similarity metric, although a number of conversions are provided. If no attribute is provided, a default weight of 1 is assigned to each edge in the network. Network clustering algorithms provide the option to set an edge weight cut-off, either by entering a value, viewing the histogram of values and using a slider to select the cutoff, or by a heuristic based on the histogram [39]. The detailed parameters for each algorithm are documented in the original papers or on the *clusterMaker *web site at: http://www.rbvi.ucsf.edu/cytoscape/cluster/clusterMaker.html.

### Algorithm-specific implementation details

Each of the algorithms provided by *clusterMaker *has been integrated into the source code to provide a consistent user interface and operation. Table 1 lists the available algorithms, with brief descriptions and implementation information.

**Table 1:** *clusterMaker *algorithm implementation notes

Algorithm	Description	Source	Details
Hierarchical	Standard hierarchical clustering as implemented by Eisen[1]	Cluster 3.0 package from Michiel de Hoon of the University of Tokyo	Ported by clusterMaker authors from C to Java

k-means	Standard k-means clustering as implemented by Eisen[1] with the addition of silhouette estimation of k	Cluster 3.0 package from Michiel de Hoon of the University of Tokyo	Ported by clusterMaker authors from C to Java. Silhouette implemented by clusterMaker authors.

k-medoid	Modification of k-means from above to use medoid rather than means		Implemented by clusterMaker authors. Silhouette implemented by clusterMaker authors.

AutoSOME	The AutoSOME cluster algorithm [34]	The distributed AutoSOME implementation	Ported directly to clusterMaker by AutoSOME author

Affinity Propagation	The message passing-based approach to clustering by Frey and Dueck[27]	Implemented from the algorithm description in the original reference	Implemented by clusterMaker authors

Connected Components	Simple division based on connectivity		Implemented by clusterMaker authors

Community (GLay)	Newman-Girvan[32] community clustering as implemented by Su, et al.[33]	The original GLay plugin for Cytoscape	Ported by clusterMaker authors

MCODE	Bader and Hogue[4] algorithm for finding modules in PPI networks	The MCODE Cytoscape plugin	Ported by clusterMaker authors

MCL	Markov clustering algorithm from van Dongen[8,28] that uses random walks to simulate flow	Implemented from original thesis with reference to C implementation for validation of results.	Implemented by clusterMaker authors as a parallel algorithm to take advantage of multiple CPU cores.

SCPS	Spectral clustering algorithm for BLAST similarity networks[24]	Implemented from the algorithm description in the original reference using the authors' implementation to validate results	Implemented by clusterMaker authors

Transitivity Clustering	Transitivity based clustering approach from Wittkop, et al.[25]	Ported from Cytoscape TransClust plugin	Ported by original TransClust authors

### Visualization

**Figure 2:**
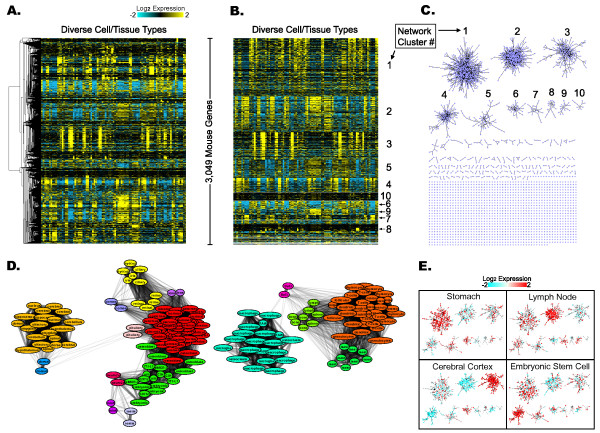
**Gene expression clustering reveals mouse protein interactome modules and fuzzy relationships among mouse cells and tissues**. Heat maps showing clusters of mouse gene expression data (GSE10246) identified using (A) hierarchical clustering and (B) AutoSOME clustering. (C) Protein interactome [45] divided into subnetworks corresponding to co-expression clusters identified by AutoSOME. (D) Fuzzy cluster network of cell/tissue types in GSE10246. Nodes represent individual cell/tissue types (labeled with first word of each sample name only), node colors correspond to different clusters, and increasing edge thickness and opacity reflect increasing frequency of co-clustering between any given pair of nodes over all ensemble iterations (see [34]). (E) Expression data of four cell/tissue types from GSE10246 superimposed onto the ten largest subnetworks from panel C (Stomach = GSM258771; Lymph Node = GSM258691; Cerebral Cortex = GSM258635; Embryonic Stem Cell = GSM258658). All expression data are log_2 _scaled and median centered. In panel B, all clusters are ordered by decreasing cluster size, and the yellow-cyan color scale is identical to panel A. In panels A and B, all arrays (cell/tissue types) are horizontally ordered the same as the GSE10246 data set.

*clusterMaker *provides three different visualizations (types of display), depending on the algorithm. Any numeric attributes within the network can be displayed as a heat map (Figure 2B). Heat maps are also used to show the results of k-means, k-medoid, and AutoSOME clustering, with each of the identified clusters separated by a bar in the heat map.

The second type of visualization, a tree view, is used by hierarchical clustering and is shown as a dendrogram combined with a heat map (Figures 1A, B, 2A). The heat map and tree view implementations were derived from Java TreeView [40], but were significantly modified to interact with the network and to function as embedded methods. Multiple heat maps or tree views may be active at the same time, allowing simultaneous display of different data sources or types. *clusterMaker*'s heat map implementations (Eisen TreeView, Eisen KnnView, and HeatMapView) all provide the ability to map colors from the heat map onto the network. This mapping can be for a single attribute in the heat map or can be used to animate through some or all of the attributes.

The third type of visualization is the network view provided by Cytoscape, but constructed by one of *clusterMaker*'s network clustering algorithms (currently Affinity Propagation, AutoSOME, Connected Components, Community, MCODE, MCL, SCPS, or Transitivity clustering). The output network shows only the intra-cluster edges (all inter-cluster edges are dropped) and the network is automatically arranged using the Cytoscape force-directed layout. The user may opt to redisplay the inter-cluster edges after the network has been laid out (Figure 1D).

All of the algorithms also provide the option of creating a Cytoscape group for each cluster. A group collects a set of nodes and their edges into one object that can be represented as a new node. For hierarchical clustering, the resulting groups are hierarchically constructed so that the user can view clustering results at any level of the dendrogram (Figure 1C - left side).

Selections in each view are linked across views. Selecting a node in Cytoscape will show that node in all of the currently displayed views. Similarly, selecting a node or group of edges in a view will select that node or group of edges in the current network, which will, in turn, update all other views. The user may also link multiple network views to allow for comparison between clustering algorithms or link heat maps or tree views to multiple different networks. Linked selection provides significant power to the user for exploring various data sets to corroborate computational results or formulate new hypotheses.

Cytoscape 2.8.2 with *clusterMaker *plugin version 1.10 was used for all of the analyses described here. Cytoscape is available from http://www.cytoscape.org and the *clusterMaker *plugin is available through the Cytoscape plugin manager. *clusterMaker *exports a number of Cytoscape commands to allow other Cytoscape plugins and software developers to take advantage of its features.

## Results

We explore how *clusterMaker *and Cytoscape might be used together by presenting three example research scenarios. Our focus is on the computational tools rather than on the specific data; the scenarios are based on previously published studies and the results are not meant to represent novel findings. It is also the case that both *clusterMaker *and Cytoscape are relatively sophisticated tools, with many features that may require some effort to fully master. Our intent is not to illustrate all of the features available in these tools, but rather to provide examples of how they can be applied to gain insight into scientific problems.

### Scenario 1: Analysis of Protein Expression Data

A principal goal of gene expression cluster analysis is to identify biologically meaningful groups of co-expressed genes or samples (i.e. transcriptomes) from potentially large data sets. Although downstream analysis of co-expression clusters typically involves exploration of enriched functional groups (e.g., using DAVID [41] or BiNGO [42]), another powerful analytical approach is to examine clusters for corresponding molecular interactions. Cluster analysis of data sets that integrate interaction and expression data can identify biomolecular networks with common expression patterns in a single step, and reveal both known and unexpected pathways [43].

Hierarchical clustering builds a tree that hierarchically connects every data point [1], but it does not automatically identify discrete clusters without the use of a tree cutting method (e.g. [44]). Depending upon the goals of the researcher, it may be desirable to identify discrete clusters from large data sets, especially for functional enrichment and biomolecular pathway analysis.

By contrast, AutoSOME identifies both discrete and fuzzy clusters from large data sets without prior knowledge of cluster number [34]. The latter feature is useful for exploring transcriptome clusters, for example, to show how different clusters of diverse transcriptomes relate to one another. In the following protocol, application of AutoSOME and hierarchical clustering to a combined protein interactome and gene expression data set is demonstrated, along with an anecdotal downstream analysis.

### Scenario 1 Data sources

A mouse protein interactome (SVM-network) was downloaded from the MppDB website (http://bio.scu.edu.cn/mppi/) [45]. This network is a product of extensive literature mining, prior knowledge of co-expressed genes and interacting domains, and other measures of functional and contextual relatedness. To integrate gene expression data, a whole-genome microarray data set representing diverse mouse cells and tissues [46] was downloaded from the Gene Expression Omnibus as a Series Matrix file (GSE10246; http://www.ncbi.nlm.nih.gov/geo/). This microarray data set contains 182 arrays (91 in duplicate) and 45,101 gene probes.

### Scenario 1 Protocol

After mapping of UniProtKB identifiers to official gene symbols using DAVID [41] and removal of duplicate edges, the mouse interactome was imported into Cytoscape. This network consists of 3,347 proteins and 13,088 non-redundant interactions. The GSE10246 expression array was pre-processed by mapping probe set identifiers to gene symbols and taking the highest expressed probe for each gene symbol. The resulting data were log_2_-scaled, and all genes were median-centered. These two normalization steps are generally recommended when using AutoSOME clustering, and can also be performed using the AutoSOME implementation within *clusterMaker*. Of the 21,864 unique genes in the expression data set, 3,049 genes were successfully mapped to the interaction network when imported into Cytoscape (Additional File 1).

Initially, the expression data were clustered hierarchically using the pairwise centroid linkage method and the uncentered correlation distance metric. All 182 array sources (i.e. transcriptomes) were used as input, and nodes without data were ignored. A heat map of the gene co-expression cluster results was rendered as a tree view with the yellow-cyan color scheme (Figure 2A).

Next, the same expression data were clustered with *clusterMaker's *AutoSOME implementation using Running Mode = Normal, P-value Threshold = 0.1, 50 Ensemble Runs, and Sum of Squares = 1 normalization (both genes and arrays) and the results were rendered as a yellow-cyan heat map (Figure 2B). Of 34 clusters and 14 singletons, 97% of all analyzed genes (2,958/3,049) fall into the largest 15 clusters. To map cluster results to the mouse interactome, a new network was created with inter-cluster edges removed (Figure 2C). In addition, AutoSOME fuzzy clustering was performed on all 182 arrays. Clustering was performed using Distance Metric = Uncentered Correlation, Running Mode = Normal, P-value Threshold = 0.05, 50 Ensemble Runs, and Sum of Squares = 1 normalization (both genes and arrays) identifying 16 fuzzy clusters. After setting the maximum number of edges to display in the fuzzy network to 4,000, 'Network' was selected in the Data Output section, and the fuzzy clusters were rendered by pressing 'Display' (Figure 2D). For increased legibility, the node and font sizes in Figure 2D were enlarged using VizMapper, a core Cytoscape component that allows for the creation and editing of network visual styles.

### Scenario 1 Results

Initially, 3,049 genes from the multi-tissue mouse microarray data set (GSE10246) were hierarchically clustered, and the resulting expression tree was rendered as a heat map (Figure 2A). Though complex gene co-expression patterns are evident in Figure 2A, it is not immediately obvious how to parse the dendrogram into discrete clusters for further analysis. By contrast, AutoSOME identified 34 discrete co-expression clusters and 14 singleton genes (Figure 2B). These clusters partition the mouse protein interaction network into 148 subnetworks and 1,432 singleton proteins (Figure 2C). Composed of 42% of all proteins in the analyzed interactome (1,282/3,049), the ten largest subnetworks are indicated in Figure 2C and their corresponding co-expression clusters are labeled in the heat map of Figure 2B.

Downstream analysis of the ten largest subnetworks (Figure 2C) using DAVID revealed highly significant functional enrichments for all but one subnetwork (Table 2). Subnetwork 1 is highly enriched in genes involved in endoderm and mesoderm differentiation pathways, important for diverse organs, subnetwork 2 genes are robustly associated with immune system functions, subnetwork 3 genes are highly enriched in neuronal processes, and subnetwork 4 genes in cell cycle activities (Table 2). To illustrate modularity in gene expression, expression levels for representative cells/tissues were mapped onto the ten largest networks using Cytoscape's VizMapper. As shown in Figure 2E, subnetwork expression profiles clearly distinguish the four selected cell/tissue types. Further, the results of the functional enrichment analysis strongly correlate with patterns of up- and down-regulation. For example, of the four cell/tissue types, subnetwork 3 is only up-regulated in the cerebral cortex sample, consistent with this subnetwork's enrichment in neuronal activity (Table 2).

**Table 2:** Enriched functional categories according to DAVID analysis, related to Figure 2C.

**Network Cluster No**.	No. Enriched Proteins (total)	Functional Category	Enrichment Score	Benjamini P-value
1	76 (483)	pattern specification process	34.7	8.3 × 10^-43^
	32 (483)	lung development	25.1	1.3 × 10^-18^
	55 (483)	blood vessel development	22.0	8.3 × 10^-27^
	71 (483)	skeletal system development	21.1	6.8 × 10^-38^
	32 (483)	gland morphogenesis	17.3	1.6 × 10^-22^

2	61 (339)	immune system development	28.4	1.5 × 10^-36^
	63 (339)	defense response	20.4	3.3 × 10^-28^

3	43 (182)	neuron projection	24.0	1.2 × 10^-34^
	38 (182)	transmission of nerve impulse	14.8	1.4 × 10^-27^

4	57 (106)	DNA metabolic process	36.5	1.1 × 10^-54^
	51 (106)	cell cycle	19.8	2.3 × 10^-37^

5	16 (65)	regulation of apoptosis	4.8	1.8 × 10^-5^
	10 (65)	chaperone	4.4	2.2 × 10^-7^

6	12 (34)	cell motion	8.3	1.4 × 10^-7^
	8 (34)	vasculature development	3.6	1.4 × 10^-4^

7	7 (24)	leukocyte differentiation	4.9	3.7 × 10^-6^
	5 (24)	regulation of T cell activation	3.2	1.9 × 10^-3^

8	15 (21)	visual perception	12.3	4.5 × 10^-19^
	9 (21)	eye development	9.4	4.2 × 10^-10^

9	0 (18)	no enrichment	NS	NS

10	8 (11)	DNA binding	4.7	9.9 × 10^-4^
	6 (11)	chordate embryonic development	4.1	1.8 × 10^-3^

Finally, in addition to gene co-expression analysis, AutoSOME fuzzy clustering was performed on the 182 transcriptomes, and the 16 resulting clusters are illustrated in Figure 2D. Along with discrete clusters denoted by different colored nodes, the fuzzy network shows how individual clusters and their constituents relate to one another. For example, as shown in Figure 2D, mast cells are more closely related to dendritic cells than macrophages, and neuro2a cells (neuroblastoma cells) are more like embryonic stem cells than cerebral cells. Such fuzzy cluster networks provide an alternative to the conventional hierarchical method for exploring intra- and inter-cluster relationships.

### Scenario 2: Identification of Protein Complexes

There are several challenges to finding complexes within a protein-protein interaction data set with clustering. Experimental sources of protein-protein interaction data include yeast two-hybrid (Y2H) [47,48] and split-ubiquitin [49] approaches, high-throughput mass spectrometric protein complex identification (HMS-PCI) [50] and tandem affinity purification followed by mass spectrometry (TAP-MS) [35,51]. Due to the multiplicity of approaches and the varying degrees of false positives and false negatives, it is difficult to have a high confidence in any particular cluster result. One approach to increasing confidence in the results of a clustering algorithm is to use additional independent data to corroborate the cluster selections. Besides increasing confidence in the clusters, combining data of different types and sources can provide additional insight into biomolecular interactions, regulatory mechanisms, and pathways. For example, combining putative protein-protein complex information with gene expression data can provide clues as to the role of individual proteins within a complex. For instance, differential expression in response to various stimuli might indicate a regulatory role for one or more of the proteins.

### Scenario 2 Data sources

A high-quality protein-protein interaction data set published in 2007 [52] forms the core network for this analysis. This data set combines three previously published high-throughput protein interaction data sets [35,50,51] to increase the quality and coverage of the resulting interaction network. The authors assigned a Purification Enrichment (PE) score to reflect the quality of interactions within the combined set.

Two yeast epistatic miniarray profiles (EMAPs) were also used: chromosome biology [53] and RNA processing [54] to provide genetic interaction data as a complement to the protein-protein interaction data set.

### Scenario 2 Protocol

The combined protein-protein interaction data set was imported into Cytoscape with a PE cutoff of 1.85, which corresponds to the scaled value of 0.20 used by the authors in the original data set [52]. The result is a network with 2742 genes and 16,218 interactions. The PE score was imported as an edge attribute and in addition to the gene symbol, the systematic name was imported as a node attribute (Additional File 2).

**Figure 3:**
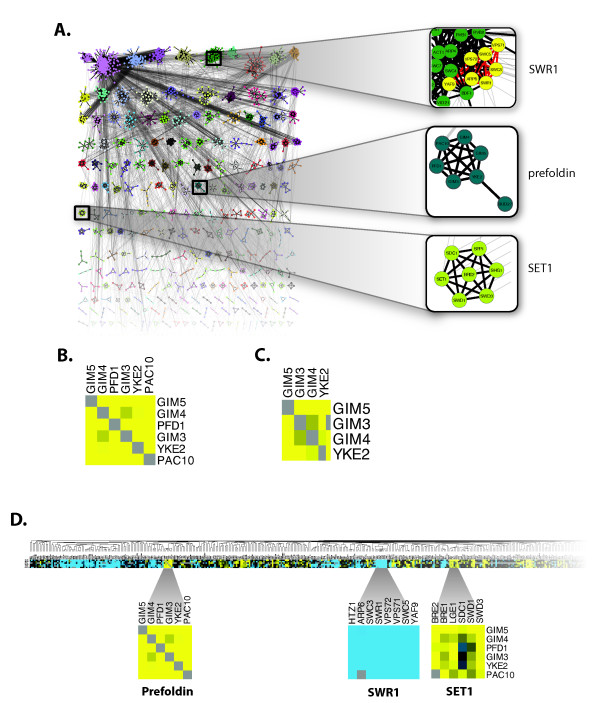
**Clustering of yeast protein-protein interaction networks in the context of overlapping yeast genetic interaction data reveals possible pathway interactions between three well-known complexes**. (A) The overall results of MCL clustering of the Collins et al., [52] data set showing the largest clusters. Nodes are colored according to cluster. Thick edges represent intra-cluster edges and thin edges are inter-cluster. Three complexes are highlighted: SWR1, SET1, and prefoldin. (B) Closeup of the prefoldin complex from the chromosome biology EMAP (Additional File 3). Note that there is a very strong positive genetic interaction (yellow) between all of the genes in the complex except for GIM3 and GIM4, which is still positive overall. (C) Closeup of the prefoldin complex from the RNA processing EMAP (Additional File 4). The closeup shows the same slightly decreased interaction for GIM3 and GIM4. (D) The section of the chromosome biology EMAP with the prefoldin complex showing the strong negative interaction with SWR1 and positive interaction with SET1.

The initial network was clustered using *clusterMaker*'s MCL implementation with the following settings: Granularity parameter = 1.8, Array sources = PE Score; and MCL Advanced Settings of: Weak edge weight pruning threshold = 1 × 10^-20^, maximum residual value = 1 × 10^-6^, and iterations = 16. MCL's iterations are not uniform, and in this example, iterations 3 and 4 take significantly more time than the other iterations. The resulting network contains 408 clusters, with the largest consisting of 254 nodes. The nodes were colored according to the cluster assignment (Figure 3A).

The EMAPs were converted into tab-delimited text files from the original Cluster (.cdt) format files with the strength of interaction imported as an edge attribute. Each EMAP was then clustered hierarchically with pairwise average linkage and uncentered correlation as the distance metric using the imported strength of interaction. The resulting clusters were shown in *clusterMaker*'s tree view with the yellow-cyan color scheme used by convention for EMAPs (Figure 3B and 3C). *clusterMaker *links selection of all heat map windows with the current network, facilitating interactive exploration and comparison of the clusters across the data sets.

### Scenario 2 Results

To explore the putative complexes derived from combining the physical interactions with genetic data, we chose the cluster formed by GIM3, GIM4, GIM5, PAC10, YKE2, and PFD, which represents the prefoldin complex.

#### Prefoldin complex

Figure 3A shows the cluster results, with the prefoldin cluster shown in the lower right. These nodes also cluster well in all of the EMAPs where they appear, particularly in the chromosome biology (Figure 3B inset) and RNA processing (Figure 3C inset) EMAPs. In each case, the interaction in the EMAPs is epistatic, which indicates that each of the pairwise double-deletion mutants grows better than might be expected given the growth rate of each single deletion mutant. An epistatic interaction is evidence that the two proteins are part of the same pathway, and the tight clustering strongly suggests that they are in the same complex. Given the results of the clustering and the strong corroboration from the genetic interaction data, it is clear that these proteins are part of the same complex. Each of these proteins is annotated in the *Saccharomyces *Genome Database (SGD) (http://www.yeastgenome.org) as part of the prefoldin complex, consistent with these results.

While this result is only confirmatory and does not provide any new knowledge about prefoldin, it is interesting to explore genetic interactions between the prefoldin complex and other putative complexes. For example, in the chromosome biology EMAP, the genes in the prefoldin complex all show a strongly negative (aggravating) genetic interaction with the genes in the SWR1 chromatin remodeling complex (APR6, SWC3, SWR1, VPS72, VPS71, SWC5, and YAF9) and a positive (epistatic) interaction with the genes in the SET1/COMPASS complex (BRE2, SWD1, and SWD3) (Figure 3D). Both SET1 and SWR1 are involved in various aspects of chromatin biology. SET1 catalyzes methylation of histone H3 and the SWR1 complex is required for the incorporation of histone variant H2AZ into chromatin. It is interesting to speculate on why SET1 and SWR1 should have opposite genetic interactions with prefoldin. This might relate to the eukaryotic specialization of prefoldin for the correct tubular assembly of actin and related tubular proteins, which are required for cell division. A role in cell division is consistent with one additional negative genetic interaction between the genes in the prefoldin complex and several of the genes involved in kinetochore-microtubule interactions (e.g. MCM16, MCM21) and tubulin folding (CIN1, CIN2, CIN4).

### Scenario 3: Protein Similarity

More than 40% of all known proteins lack any annotations within public databases [55]. As a result, millions of proteins are completely uncharacterized except for sequence and (possibly) predicted domain architectures. A number of approaches have been proposed for classifying proteins by function[7,8,11-28], and *clusterMaker *provides several algorithms well-suited for clustering proteins based on some similarity metric such as BLAST [56]. While sequence clustering approaches do not provide a definitive categorization of proteins, these approaches can be extremely useful as initial steps in an overall curation pipeline. *clusterMaker *allows researchers to rapidly cluster data sets and visualize the results. By mapping protein function annotations to visible node properties, the curator may immediately discern clusters that include both unknowns and functionally characterized proteins. The availability of multiple clustering algorithms allows the curator to assign a greater confidence to those predictions that appear consistently across multiple clustering outputs. This approach can significantly reduce the overall curation timeline, particularly in the early stages before other approaches such as hidden Markov models (HMMs) are applicable.

### Scenario 3 Data sources

The Structure-Function-Linkage Database (SFLD) is a gold-standard resource tool linking sequence information from mechanistically diverse enzyme superfamilies to their characterized structural and functional properties [57]. The SFLD provides a three-level classification for proteins: superfamily - evolutionarily related proteins that catalyze the same partial reaction, family - proteins within a superfamily that catalyze the same overall reaction, and subgroup - a mid-level classification containing multiple families with shared functional residue motifs.

From the superfamilies present in the SLFD, we chose to cluster the vicinal oxygen chelate (VOC) superfamily, a group of metal-dependent enzymes that share a common fold motif and catalyze a variety of reactions [58]. It is difficult to discriminate specific functions (overall reaction catalyzed and thus family membership) within this superfamily due to multiple, perhaps serial permutations and other rearrangements in its evolutionary history [59]. The VOC superfamily data set is composed of 683 protein sequences, partially classified among seven subgroups and 17 families. Less than half of these sequences included both a family and subgroup classification, and 224 sequences contained a subgroup classification but not a family classification. The remaining 168 sequences were completely uncharacterized.

### Scenario 3 Protocol

The SFLD VOC superfamily was loaded into Cytoscape through the SFLDLoader plugin with an e-value cutoff of 1e^-1^(Additional File 5). Nodes in the network represent individual proteins, with family and subgroup classifications already specified among the properties of the nodes. Edges in the network represent protein similarities based on the BLAST e-values of each pairwise sequence alignment.

**Figure 4:**
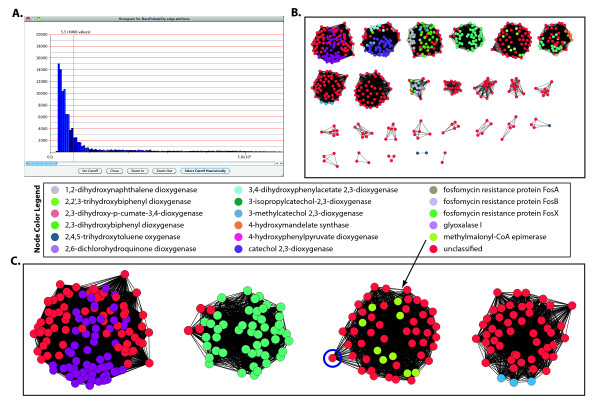
**Protein similarity network clustering indicates possible family membership for uncharacterized proteins**. (A) A distribution of edge weights (binned -log(BLAST E-values)) of the VOC superfamily is shown, with a cutoff value of 5.5 indicated by a red vertical line. The cutoff was determined by a heuristic described in [53] and was used for subsequent clustering. (B) MCL clusters for the VOC superfamily are displayed with nodes colored by family assignment. Red nodes represent proteins with unknown function. (See Additional File 6 for TransClust Clusters). (C) Four clusters within the MCL clustering results show only proteins from a single family or proteins of unknown function. (Three of these four clusters also appear in the TransClust results.) Based on this analysis, we hypothesize that the function of the unknowns is the same as that of the other proteins in each cluster. The protein highlighted in blue is BH2212, which was randomly selected for further analysis.

*clusterMaker *was used to select a cutoff based on properties of the network edge weight distribution (Figure 4A). This cutoff selection heuristic has been shown to improve the accuracy of clustering a protein similarity network into families [39]. Using the -LOG(value) edge weight conversion, a heuristically determined cutoff of 6.0 was used for all clustering runs.

MCL, TransClust and SCPS clustering were performed on the VOC protein similarity network. Default parameters were used except that the MCL granularity parameter was set to 2.5. Clustering outputs were visualized by coloring the nodes based on known family assignments (where available), allowing rapid identification of clusters composed of characterized members of a single family plus uncharacterized nodes. Such uncharacterized nodes are potential members of the co-clustered family.

### Scenario 3 Results

MCL generated 26 clusters and TransClust generated 28 clusters. These numbers adequately approximate the presence of 17 distinct families in 50% of the VOC data set. SCPS, on the other hand, generated only three clusters, which indicates an overabundance of false positives in the SCPS clustering data. Therefore, further analyses focused only on the MCL and TransClust clustering results. As shown in Figure 4B, these results are dominated by uncharacterized proteins (colored red in the figure). Certain clusters are composed entirely of uncharacterized proteins, while other clusters are composed of uncharacterized proteins as well as two or more known families. The most interesting clusters contain just two colors, representing the grouping of uncharacterized proteins with a single VOC family. These clusters allow us to hypothesize the functions of the uncharacterized proteins.

Three such single-family clusters are present in almost equal measures across both the TransClust and MCL results (Figure 4C), one of which is the methylmalonyl-CoA epimerase subgroup of 50 proteins (arrow in Figure 4C). This includes the nine characterized members of the methylmalonyl-CoA epimerase family and 41 sequences that lack a family classification in the SFLD, although they are annotated to be in the same subgroup. The size of the cluster is 52 in the TransClust results and 53 in the MCL results. The additional few nodes represent sequences lacking a subgroup classification and that appear in both the TransClust and MCL results, suggesting that putatively assigning these to the methylmalonyl-CoA epimerase subgroup would be reasonable.

In an effort to seek additional evidence of family and subgroup membership, we explored in some detail a randomly chosen uncharacterized protein on the periphery of the methylmalonyl-CoA epimerase cluster (see Figure 4C). The hypothetical (predicted) protein BH2212 from *Bacillus halodurans *(gi:15614775) lacks both a family and subgroup assignment. We aligned its sequence with that of methylmalonyl-CoA epimerase from *Propionibacterium shermanii *(gi:15826388). Four of the five functionally critical active site residues align perfectly with the uncharacterized sequence. These four residues bind the active-site metal ion needed for catalysis. In the initial alignment, the fifth residue, a glutamic acid that abstracts a proton from the substrate, is shifted by one position, but minor editing can also align this residue without degrading the rest of the alignment. Thus, the unknown protein is likely capable of binding the active site metal and may also perform the epimerization of (2R)-methylamonyl-CoA. The next step in functional annotation of this sequence would be to compare it to the hidden Markov models (HMMs) used to characterize the methylmalonyl-CoA epimerase family and subgroup in the SFLD or experimentally verify the function of the protein. These further analyses are beyond the scope of this paper.

## Discussion

*clusterMaker *is not the first package to combine a number of clustering algorithms with several viewing options. The excellent MeV package [60,61], which is part of the TM4 microarray analysis package, provides clustering algorithms and visualizations for analyzing microarray data. But *clusterMaker*, while providing fewer microarray analysis algorithms and visualizations than MeV, adds a relatively simple and consistent user interface together with the ability to interconnect multiple types of data (expression, genetic interaction, physical interaction) interactively, and to combine the power of cluster analysis with network analysis.

Such interconnections and combinations may provide additional confidence in the results, as some of the clustering methods complement one another, or simply a more in-depth exploration of the data. For example, the hierarchical and MCL clusters agree well in scenario 2, but the hierarchical heat map visualization shows the additional neighborhood context around the clusters. This context might be useful to show potential temporal interactions, or proteins that might be shared between complexes. Similarly, the use of multiple approaches in scenario 1 provides very different views of the data which can highlight relationships and groupings not obvious in any single view, and using multiple clustering approaches in scenario 3 improves our confidence in putative functional assignments.

A key feature of *clusterMaker *is the ability to link results across all views, whether heat map or network. This interactive linking is a critical aspect of the design and implementation of *clusterMaker *and allows researchers to explore data in a number of different ways without having to remember results or manually compare values.

*clusterMaker *is designed to be part of the Cytoscape environment. First, all of the clustering algorithms allow users to create Cytoscape groups that may be used by other Cytoscape plugins for further analysis, or by users to select all of the members of a given cluster or to collapse an entire cluster into a "meta node". Second, all of the algorithms store their results as Cytoscape attributes that are available to other plugins and saved with a Cytoscape session. Finally, *clusterMaker *exports all of its algorithms and visualizations for use by other plugins through the CyCommand API provided in Cytoscape. This provides a mechanism for other plugin developers to take advantage of *clusterMaker*'s capabilities improving overall reuse. Through the Cytoscape commandTool plugin, users may script clusterMaker's clustering actions and visualizations through a command file.

Several improvements to *clusterMaker *will be implemented in the future. First, we plan to add a number of algorithms to *clusterMaker*, including HOPACH [62], Quality Threshhold [63], as well as fuzzy c-means [64] or other fuzzy clustering approaches. Second, additional pre-clustering and post-clustering filter options could be incorporated, such as the Fluff, and K-Core filtering options used in MCODE [4] or the Best neighbor methods provided by jClust [65]. Third, coupling enrichment analysis such as BiNGO [42] with clustering results could be very useful. Finally, there are several additional visualization options that might be added, including the addition of one-dimensional histograms to the tree and heatmap views, visual identification of clusters formed by selecting dendrogram cutoffs, interactive setting of parameters, and many others. We believe the needs of *clusterMaker *users and shifting biological data sets should be the primary driver in *clusterMaker*'s evolution, so it is likely that as *clusterMaker *evolves other algorithms and visualizations will be added to the list.

## Conclusions

*clusterMaker *is an important addition to the suite of Cytoscape plugins. It provides a clustering framework that allows users to compute and visualize clusters in multiple ways and interactively explore the results across all of the various approaches. *clusterMaker's *algorithms include several unique additions to Cytoscape, including hierarchical clustering, k-means and k-medoid clustering, AutoSOME, SCPS, and a multi-threaded Java implementation of MCL. It also adds to these unique algorithms unique visualizations including heatmaps with (TreeView) or without dendrograms (HeatMap, KnnView), clustered network views, and clustered network views with inter-cluster edges. Using *clusterMaker*, all of these visualizations may be linked together to support interactive exploration of the data sets. All of these algorithms and visualizations are available to be used by other Cytoscape plugins or through command scripts. These capabilities allow researchers to interactively explore, analyze and compare a variety of different data within a network context. We will be adding additional algorithms and visualizations to meet new clustering requirements as they arise.

## Availability and requirements

**Project name: **clusterMaker

**Project home page: **http://www.rbvi.ucsf.edu/cytoscape/cluster/clusterMaker.html

**Installation**: *clusterMaker *1.10 is available from the Cytoscape Plugin Manager under the **Analysis **category

**Source**: http://chianti.ucsd.edu/svn/csplugins/trunk/ucsf/scooter/clusterMaker

**Operating system(s): **Platform independent

**Programming language**: Java

**Other requirements**: Java 1.6 or higher, Cytoscape 2.8.2 or higher

**License: **GNU GPL

**Any restrictions to use by non-academics: **None

## Authors' contributions

JHM wrote *clusterMaker *and performed the Scenario 2 analysis. LA added heuristic cutoffs, SCPS, and AP algorithms to *clusterMaker *and performed the Scenario 3 analysis. AMN added AutoSOME to *clusterMaker *and performed the Scenario 1 analysis. JB and TW contributed Transitivity Clustering to *clusterMaker*, GS contributed GLay to *clusterMaker*, and GDB contributed MCODE to *clusterMaker*. TEF supervised the overall implementation of *clusterMaker *and the design of the scenarios. All authors read and approved the final manuscript.

## Supplementary Material

Additional file 1 **Cytoscape session file for scenario 1**. A Cytoscape session file contains a network with the mouse protein-protein interaction data set discussed in scenario 1 as well as the imported expression data. Click here for file

Additional file 2 **Cytoscape session file for scenario 2**. A Cytoscape session file contains a network representing the Collins, et al. data set as well as two of the EMAPs discussed in scenario 2. Click here for file

Additional file 3 **Chromosome biology EMAP**. Results of the *clusterMaker *hierarchical cluster of the chromosome biology [53] EMAP. Click here for file

Additional file 4 **RNA processing EMAP**. Results of the *clusterMaker *hierarchical cluster of the RNA processing [54] EMAP. Click here for file

Additional file 5 **Cytoscape session file for scenario 3**. A Cytoscape session file with the VOC superfamily downloaded from the Structure-Function Linkage Database. Click here for file

Additional file 6 **TransClust results**. Results of clustering the VOC superfamily using *clusterMaker*'s Transitivity Cluster implementation. Click here for file
