# Supplementary figures and images for: clusterMaker: a multi-algorithm clustering plugin for Cytoscape
Source: BMC Bioinformatics. 2011 Nov 9;12:436. doi: 10.1186/1471-2105-12-436 (PMC3262844; doi:10.1186/1471-2105-12-436)

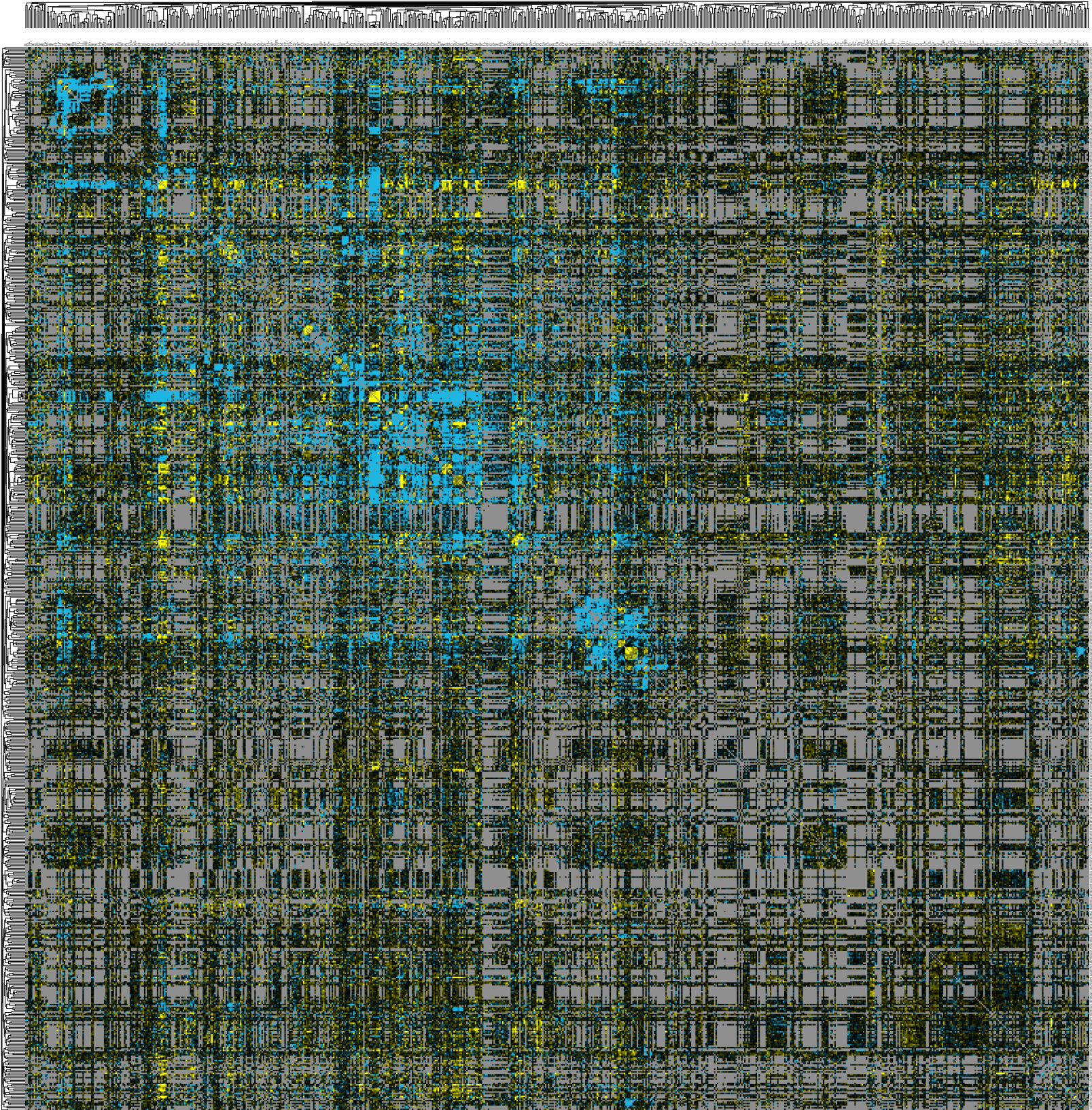

Supplement: Additional file 3 — Chromosome biology EMAP. Results of the clusterMaker hierarchical cluster of the chromosome biology [53] EMAP. [file 1471-2105-12-436-S3.PDF]

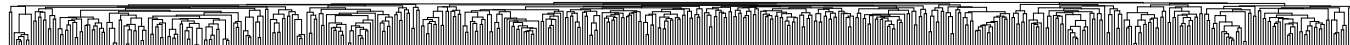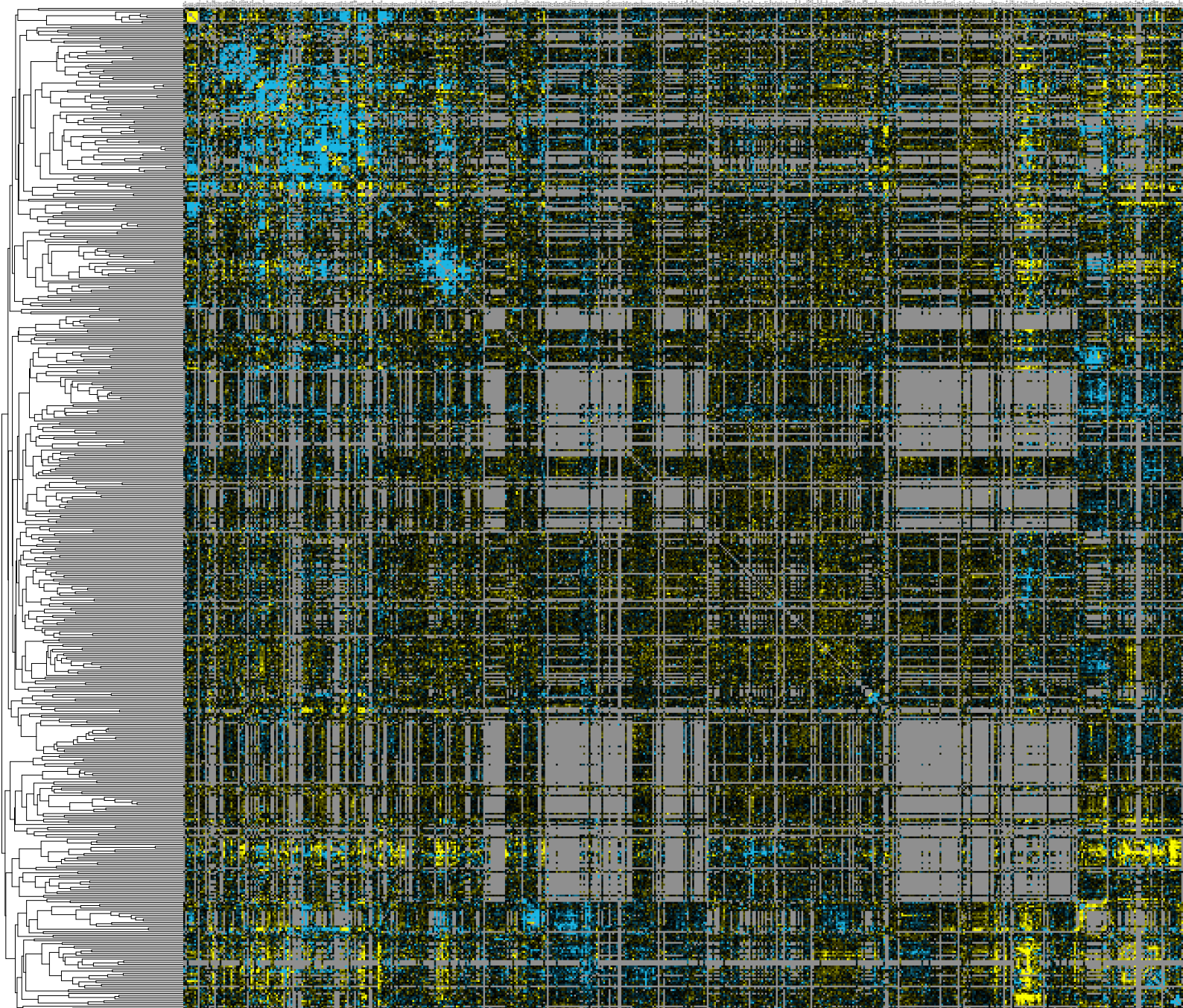

Supplement: Additional file 4 — RNA processing EMAP. Results of the clusterMaker hierarchical cluster of the RNA processing [54] EMAP. [file 1471-2105-12-436-S4.PDF]

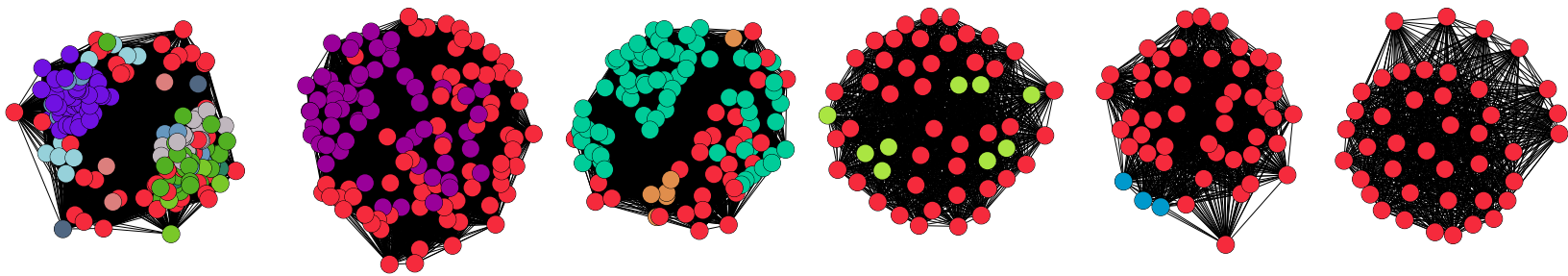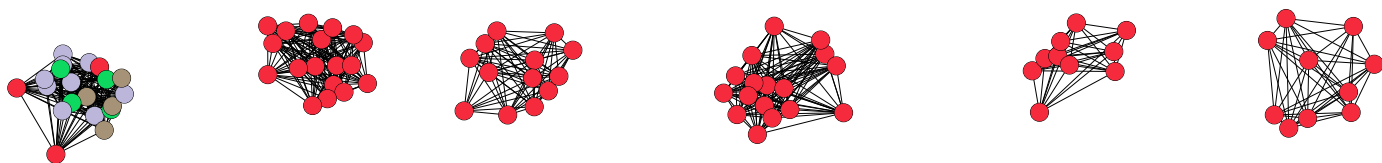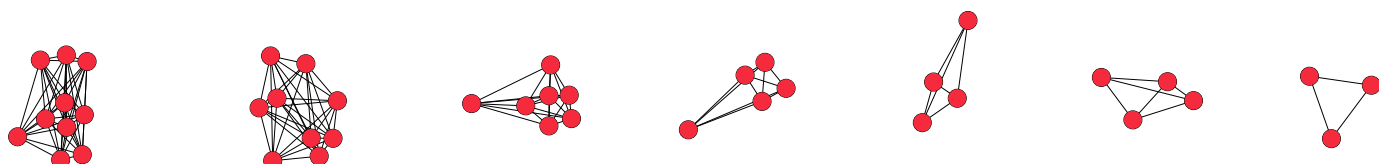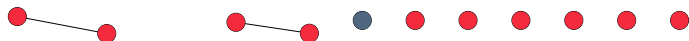

Supplement: Additional file 6 — TransClust results. Results of clustering the VOC superfamily using clusterMaker's Transitivity Cluster implementation. [file 1471-2105-12-436-S6.PDF]
